# Learning Effects in Air Displacement Plethysmography

**DOI:** 10.3390/life13061315

**Published:** 2023-06-02

**Authors:** Paul Muntean, Anca Popa, Monica Miclos-Balica, Falk Schick, Oana Munteanu, Vasile Pupazan, Adrian Neagu, Monica Neagu

**Affiliations:** 1 Department of Functional Sciences, ”Victor Babeș” University of Medicine and Pharmacy of Timișoara, 300041 Timisoara, Romania; muntean.paul@umft.ro (P.M.); anca.lp@gmx.com (A.P.); moni.0912@yahoo.com (M.M.-B.); falk_schick@web.de (F.S.); oanamnt@gmail.com (O.M.); pupazan.vasile@umft.ro (V.P.); neagu.monica@umft.ro (M.N.); 2 Center for Modeling Biological Systems and Data Analysis, ”Victor Babeș” University of Medicine and Pharmacy of Timisoara, 300041 Timisoara, Romania; 3 Department of Rehabilitation, Physical Medicine and Rheumatology, Research Center for Assessment of Human Motion, Functionality and Disability, ”Victor Babeș” University of Medicine and Pharmacy of Timișoara, 300041 Timisoara, Romania; 4 Department of Physics and Astronomy, University of Missouri, Columbia, MO 65211, USA

**Keywords:** BOD POD, reliability, body volume, body fat percentage, fat-free mass

## Abstract

Air displacement plethysmography (ADP) is a widespread technique for assessing global obesity in both health and disease. The reliability of ADP has been demonstrated by studies focused on duplicate trials. The present study was purported to evaluate learning effects on the reliability of body composition assessment using the BOD POD system, the sole commercially available ADP instrument. To this end, quadruplicate trials were performed on a group of 105 subjects (51 women and 54 men). We estimated measurement error from pairs of consecutive trials—(1,2), (2,3), and (3,4)—to test the hypothesis that early measurements are subject to larger errors. Indeed, statistical analysis revealed that measures of reliability inferred from the first two trials were inferior to those computed for the other pairs of contiguous trials: for percent body fat (%BF), the standard error of measurement (SEM) was 1.04% for pair (1,2), 0.71% for pair (2,3), and 0.66% for pair (3,4); the two-way random effects model intraclass correlation coefficient (ICC) was 0.991 for pair (1,2), and 0.996 for pairs (2,3) and (3,4). Our findings suggest that, at least for novice subjects, the first ADP test should be regarded as a practice trial. When the remaining trials were pooled together, the reliability indices of single ADP tests were the following: ICC = 0.996, SEM = 0.70%, and minimum detectable change (MDC) = 1.93% for %BF, and ICC = 0.999, SEM = 0.49 kg, and MDC = 1.35 kg for fat-free mass (FFM). Thus, the present study pleads for eliminating learning effects to further increase the reliability of ADP.

## 1. Introduction

Monitoring nutritional status beyond surrogate measures, such as the body mass index (BMI) or girths, has gathered momentum recently, mainly because of the emergence of new techniques able to characterize various components of the human body. Furthermore, increasing evidence suggests that surrogate measures can be misleading as predictors of cardiometabolic disease risks [1]. In the simplest model, our body is considered to be made of two major components: body fat (mainly triglycerides) and fat-free mass [2,3]. Tracking the fat-free mass (FFM), as opposed to body mass (BM) or BMI, is key to body weight management. Indeed, mere caloric restriction has highly variable outcomes in different individuals and results in a reduction in both fat mass (FM) and FFM. Typically, 25% of weight loss results from losing FFM, but the inter-individual variance is high, and, in some cases, this fraction can exceed 50% [1,4]. Losing body fat is a common goal for patients who suffer from the side effects of obesity [1], for athletes who practice weight-sensitive sports [5], and generally, for people interested in a healthy lifestyle. A common method for assessing the amount of body fat is densitometry. The person is weighed, their body volume (BV) is measured, and body density (D = BM/BV) is calculated. Then, population-specific formulas express body fat percentage (%BF, defined as (FM/BM) × 100%) in terms of body density [6]. Thus, the determination of fatness by densitometry hinges on accurate measurements of BV.

Body volume can be measured by hydrostatic weighing (HW) [7], as well as by air displacement plethysmography (ADP) [8,9]. Both are noninvasive, but the latter is more convenient because it is faster and less burdensome from the subject’s point of view. HW has long been the gold standard of densitometry, and ADP has been calibrated to provide excellent agreement with HW for the adult population [9]. Since the development of the first commercially available ADP system, called BOD POD^®^ (COSMED, Concord, CA, USA), the validity of ADP relative to HW has been assessed by numerous studies, which reported standard errors of the estimate ranging from 1.8% to 2.3% BF in adults and 3.3% BF in children [10]. ADP and HW were also in very good agreement in the geriatric population; on average, ADP overestimated body fatness by 0.8 %BF [11]. Studies stratified by sex found that ADP overestimated %BF in women (by roughly 1 %BF, on average) [10,12] and underestimated it in men to the same extent [12]. ADP is also used, alongside other techniques, to quantify multiple components of the human body. The criterion four-component (4C) model, for example, relies on ADP or HW to measure BV, deuterium dilution to assess total body water, and dual-energy X-ray absorptiometry (DXA) to measure the bone mineral content [13].

ADP has several advantages over other techniques of body composition analysis. It is user- and subject-friendly, noninvasive, and feasible for infants, children, youth, and adults [14]. HW is an equally accurate method to assess body fat at the molecular level, but it takes more time and is more demanding on the subject. DXA is highly regarded because it is accurate, precise, and capable of describing the regional distribution of adipose tissue and bone mineral. On the other hand, DXA should be used sparingly (at most four times per year) because it exposes the subject to X-rays. Field methods, such as anthropometry, A-mode ultrasound, and bioelectrical impedance analysis, are less accurate in evaluating the total amount of body fat, but they are less expensive and portable [5].

The accuracy of ADP has been evaluated against a variety of techniques of body composition assessment [10,15], and it continues to attract much research effort [6,16,17,18,19]. Less attention has been paid, however, to an equally important problem, the reliability of ADP. Reliability refers to the ability of an instrument to provide measurement results close to one another. Ideally, an instrument should be both accurate and reliable—i.e., it should indicate the true value of the measured quantity and do it reproducibly. Nevertheless, even with limited accuracy, a highly reliable instrument can be used to track changes in the measured quantity. For example, despite significant differences between body composition measures given by ADP and DXA at any given time point, both techniques indicated similar changes in %BF and FFM during a weight loss intervention [20]. The present study does not deal with the validity of ADP; it is designed to evaluate its reliability in a chain of successive pairs of measurements.

The reliability of ADP has also been scrutinized in several works [9,21,22,23,24,25,26,27,28,29,30], which culminated in the study of Noreen and Lemon [27], conducted on a sample of 980 subjects spanning a wide range of body compositions. In %BF measurements, they determined an intraclass correlation coefficient of 0.996 and a technical error of measurement of 1.07% BF, which led them to conclude that ADP is a reliable measure of global adiposity. Nevertheless, with few exceptions [21,26,30], previous reliability studies focused on duplicate trials, and, to our knowledge, none of them looked into learning effects.

In a test involving human subjects, learning effects consist of a systematic change in the outcome of the test when it is administered repeatedly. The subject becomes acquainted with the test procedure and modifies their attitude (intentionally or not) to change the test’s result (e.g., obtain a better score in a physical performance assessment). A learning effect may originate from a change in skill, motivation, fatigue, or anxiety caused by the previous trial [31]. To uncover learning effects, one needs to run three or more contiguous trials on each participant and calculate statistical measures of reliability for consecutive pairs of trials—(1,2), (2,3), (3,4), and so on. If learning effects are present, it is important to determine how many practice trials are needed before the reliability levels off. Then, consecutive trials with similar standard errors of measurement can be analyzed together for a more precise evaluation of the reliability of individual trials. Based on simulations, Hopkins established that a precise estimate of the change in the standard error of measurement requires at least 50 subjects [32].

The present study was designed to evaluate the hypothesis that body composition assessments by ADP might be affected by learning effects stemming from the gradual accommodation of the subject to the test procedure. If learning effects indeed play a sizable role in ADP, the question is how to minimize their impact on body composition assessments. A second objective of this study was to evaluate the technical error of measurement, standard error of measurement, and minimal detectable change in ADP tests when practice trials are discarded.

## 2. Materials and Methods

This study was performed under the ethical guidelines of the Declaration of Helsinki. Before being enrolled, each volunteer signed a form of informed consent. We explained the physical principles of ADP to each potential participant, emphasizing that the measurement is not painful and does not involve harmful physical factors. The study protocol was approved by our institutional Committee of Research Ethics (resolutions no. 20/24 July 2019 and 42/2 June 2022).

### 2.1. Subjects

We recruited volunteers via social media and flyers distributed locally. Potential participants satisfied all of the following inclusion criteria: (i) age between 18 and 70 years, (ii) no experience with ADP tests, and (iii) willingness to wear minimal clothing recommended by the manufacturer of the BOD POD [33]. Candidates were not enrolled if they met any one of the following exclusion criteria: (i) health record with chronic diseases or (ii) diagnosed with acute infections. Table 1 presents the characteristics of the study participants (51 women and 54 men). The sequence of ADP measurements needed for this study was completed by all the enrolled participants (i.e., there were no dropouts).

### 2.2. ADP Measurements

Each day of measurements commenced with a system quality check and scale calibration.

To prepare for ADP assessments, participants were asked not to drink or eat during the previous 4 h. They were also asked to use the restroom (void their bladder and/or bowel) right before the first test.

First, height was measured in triplicate, to the nearest 0.5 cm, using a GIMA 27335 wall-mounted tape measure (GIMA, Gessate, Italy), with the subject’s Frankfort plane in the horizontal position. The median of stature measures was entered into the BOD POD software and used to predict the subject’s thoracic gas volume (based on age, sex, and height) [33].

Then, four to six ADP trials were performed on each subject, in close succession, using a BOD POD Gold Standard Body Composition Tracking System (COSMED USA, Concord, CA, USA) with software version 5.3.2 [33]. The first four of them were included in this investigation of learning effects.

ADP tests were performed by operators with at least one year of experience with the BOD POD. Tests were conducted according to the instructions provided by the instrument’s software. Each trial involved at least two body volume measurements of about 50 s duration each. If they were within 150 mL of each other, the software computed their mean for further calculations. Otherwise, it instructed the operator to perform a third measurement, and the mean of the two closest results was used by the software to compute body composition variables [33]. Rarely, when none of the three measurements are consistent with each other, the software suggests repeating the entire trial—this situation was not observed in the present study.

During the test, the subject wore a form-fitting Lycra^®^ or spandex swimsuit or single-layer compression shorts and a jog bra. A Lycra^®^ swim cap was also worn to minimize isothermal air pockets near the scalp; it was put on before the first trial and kept for the entire set of measurements. Jewelry and/or glasses were removed by the subject before being tested.

Consecutive trials were carried out from scratch, with the repositioning of the subject after each trial. Special care was taken, however, to ensure that the subject adopted a well-defined position each time, without touching the backrest, with legs slightly apart and hands on the knees. Asking for a standard position has been proposed previously to minimize variability [34,35].

### 2.3. Statistical Analyses

In this paper, we represented experimental data using violin plots [36]. The Shapiro–Wilk test was used to assess normality. In the absence of normality, the Kruskal–Wallis test was applied to determine whether data sets collected in successive trials come from the same distribution. The level of statistical significance was set to 0.05.

To characterize the test-retest reliability of body composition assessments by ADP, we performed Bland–Altman (BA) analyses and computed several statistical measures of reliability for consecutive pairs of trials.

In a BA analysis, the differences, $d_{i}$, of two scores (body composition variables) recorded in successive trials are plotted against their mean; i=1,2,…,n labels study participants and n is the sample size. The mean value of the differences, $\bar{d}$, called bias, is plotted as a solid horizontal line, and it is flanked by the 95% limits of agreement, $\bar{d}\pm 1.96\;\mathrm{SD}$, depicted as dashed horizontal lines—here, SD denotes the standard deviation of differences, and 1.96 is the *z* score that corresponds to a 95% level of confidence. The 95% confidence intervals (CI) of the bias and the limits of agreement are shown as vertical segments (error bars) centered on the corresponding horizontal lines [37,38,39].

We applied Dahlberg’s formula to compute the technical error of measurement (TEM) inferred from pairs of trials: $\mathrm{TEM}=\sqrt{\left(\sum_{i=1}^{n}d_{i}^{2}\right)/\left(2n\right)}$ [40,41].

As relative measures of reliability [42], we computed the 2-way random effects model intraclass correlation coefficient, ICC (2,1) [43], and Lin’s concordance correlation coefficient (CCC) [44]. We also conducted a 2-way analysis of variance (ANOVA)—with trials as the primary factor and subjects as the secondary factor—to calculate the standard deviation of the scores from all subjects, ${\mathrm{SD}}_{\mathrm{all}}=\sqrt{{\mathrm{SS}}_{\mathrm{total}}/\left(kn-1\right)}$, where ${\mathrm{SS}}_{\mathrm{total}}$ is the total sum of squares, and $\left(kn-1\right)$ is the number of degrees of freedom when k trials are performed on each subject. Then, the standard error of measurement (SEM) was estimated as $\mathrm{SEM}={\mathrm{SD}}_{\mathrm{all}}\sqrt{1-\mathrm{ICC}\left(2,1\right)}$ [42].

Finally, we also computed the minimum detectable change, $\mathrm{MDC}=1.96\;\sqrt{2}\;\mathrm{SEM}$, defined as the smallest difference between two scores that reflects an actual change rather than a random variation due to measurement error [45]. Here, the factor $\sqrt{2}$ takes into account that both scores were measured with error. MDC is also known as the minimum difference needed to be considered real [42].

For statistical computations and graphics, we used MedCalc version 20.015 (MedCalc Software Ltd., Ostend, Belgium).

## 3. Results

**Figure 1:**
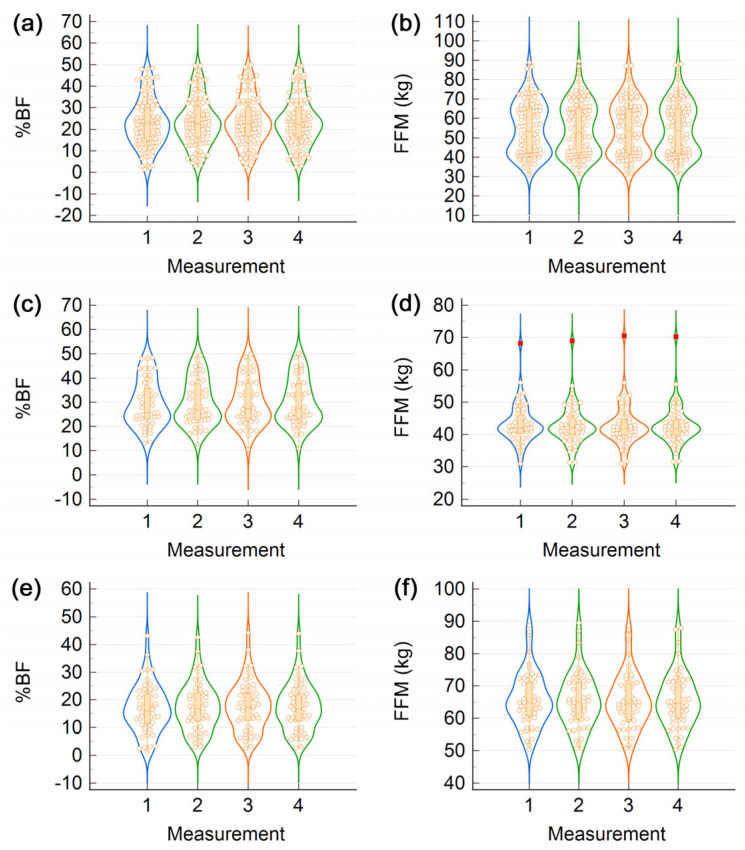
Violin plots of data recorded in four successive ADP tests. Individual panels represent (**a**) the body fat percentage (%BF) of all participants, (**b**) the fat-free mass (FFM) of all participants, (**c**) %BF of women, (**d**) FFM of women, (**e**) %BF of men, and (**f**) FFM of men.

Body composition data acquired in four successive measurements are characterized by the violin plots shown in Figure 1. In each plot, empty circles represent individual data points. The box from the center is delimited by the first quartile (Q_1_) and third quartile (Q_3_) (bottom and top margins, respectively) and divided by the second quartile (Q_2_), also known as the median; 25% of the data points reside below Q_1_, 50% lie below the median, and 75% lie below Q_3_. The height of the box is a measure of the interquartile range (IQR = Q_3_ − Q_1_). Vertical lines protrude from the box down to the lower adjacent value (the lowest value just above Q_1_ − 1.5 IQR) and up to the upper adjacent value (the highest value just below Q_3_ + 1.5 IQR). Data points beyond the lines are considered outliers: those within 3 IQR from the margins of the box are known as outside values, whereas those that lie even farther are called far-out values and are represented by different markers (see, e.g., red squares in Figure 1d) [46]. The lateral profile of a violin plot is symmetric with respect to the vertical axis because each side is a graphical representation of the probability density function (a smoothed histogram). Hence, the width of the violin plot shows how often the given value is encountered in the data set [36].

When the entire data set was analyzed, %BF displayed a unimodal distribution (Figure 1a), whereas FFM had a bimodal distribution (Figure 1b). For each sex, %BF and FFM had unimodal distributions (Figure 1c–f), but the peaks of the corresponding probability density functions (the modes) of women were shifted with respect to those of men. Interestingly, for %BF and FFM, outliers (data points beyond the whiskers) were present for each sex in part (Figure 1d–f), but not in the entire data set (Figure 1a,b). Figure S1 from the Supplementary Materials shows violin plots of body volume (BV) for the entire sample (Figure S1a), for women (Figure S1b), and for men (Figure S1c).

The lateral profiles of the violin plots from Figure 1 and Figure S1 deviate from the normal probability density function. Indeed, the *p*-values of the Shapiro–Wilk test indicate that none of the data sets were normally distributed (Table S1).

**Figure 2:**
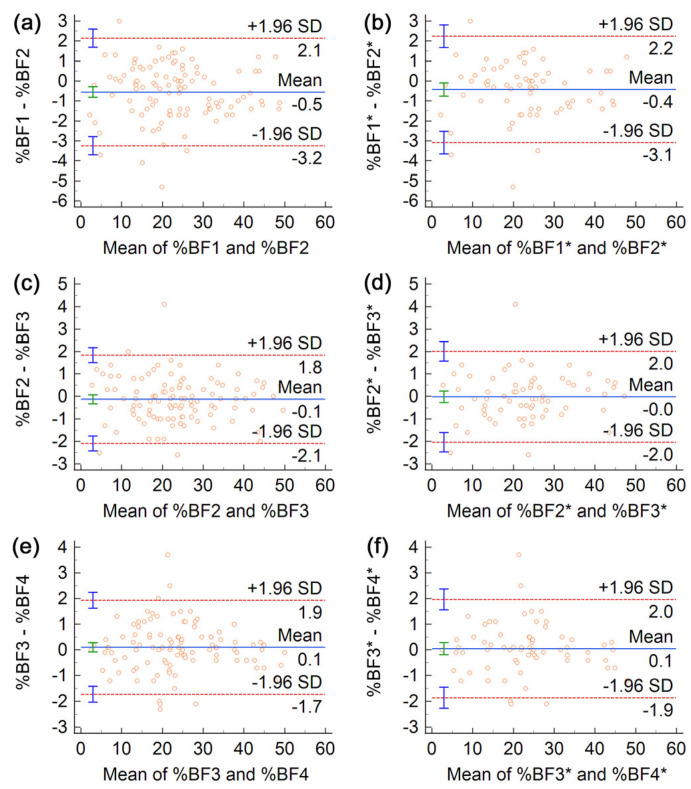
Bland-Altman (BA) plots of differences vs. means of %BF obtained in pairs of consecutive trials. The outcomes of the first two trials are compared in (**a**,**b**), trials 2 and 3 are compared in (**c**,**d**), and trials 3 and 4 are compared in (**e**,**f**). Panels (**a**,**c**,**e**) refer to the entire data set, whereas (**b**,**d**,**f**) do not include participants evaluated at the beginning of the day of measurements (an asterisk, *, labels body composition variables of subjects who did not start off the testing day). In each BA plot, the blue solid line represents the bias, the red dashed lines represent the limits of agreement, whereas the error bars depict the 95% confidence intervals of the corresponding statistical parameters.

We also examined whether there were statistically significant differences between successive body composition assessments. Therefore, we performed the Kruskal–Wallis test to evaluate the null hypothesis that the four data sets come from the same distribution. The corresponding *p*-values were larger than 0.05 (Table S2), casting no doubt on the validity of the null hypothesis. Nevertheless, despite the absence of statistically significant differences between the results of contiguous trials, the Bland–Altman (BA) plots from Figure 2 indicate that, on average, the first trial deviates from subsequent ones by 0.4% to 0.6% BF, providing a slight underestimation of the subject’s adiposity. Indeed, the horizontal line labeled “Mean” in Figure 2a indicates a bias of −0.5% BF—that is, compared to the first trial, the second trial provided higher body fat estimates by 0.5% BF, on average. Moreover, this bias is statistically significant because (unlike in Figure 2c,e) in Figure 2a zero does not belong to the 95% CI of the bias (represented as a green error bar).

We next asked the question of whether the discrepancy between the first assessment and subsequent ones could stem from insufficient warmup of the BOD POD in the course of the initial quality check procedure recommended by the manufacturer [33]. Therefore, in Figure 2b,d,f, we excluded firstcomers (21 women and 17 men) from the BA analysis. The similarity of panels (a,b), (c,d), as well as (e,f), suggests that the significant bias observed between the first two trials cannot be ascribed to warmup issues. A comparison of Table 2 and Table S3 conveys the same conclusion.

**Figure 3:**
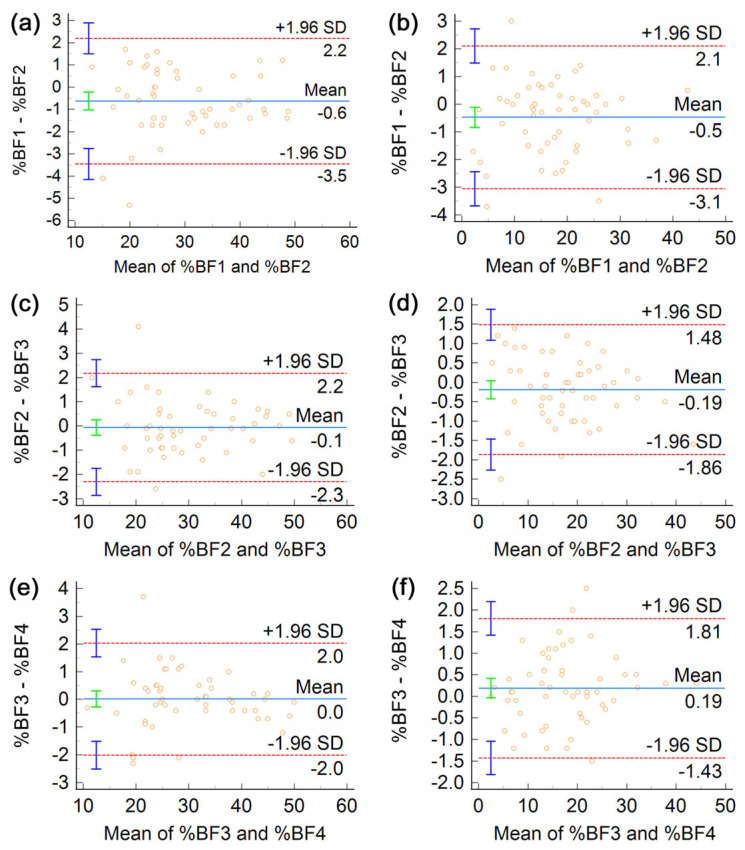
BA analysis of learning effects stratified by sex. Trials 1 and 2 are compared in (**a**,**b**), trials 2 and 3 are compared in (**c**,**d**), and trials 3 and 4 are compared in (**e**,**f**). Panels (**a**,**c**,**e**) are BA plots of %BF estimates of women, whereas (**b**,**d**,**f**) are the corresponding plots obtained for men.

Figure 3 presents BA plots meant to evaluate learning effects in ADP tests in women and men separately.

The BA analysis of FFM assessments (Figure S2a,c,e) indicates a significant bias of 0.4 kg between the first and second trial (an overestimation of FFM in trial 1 compared to trial 2) and negligible bias for pairs (2,3) and (3,4). Furthermore, the bias between BV measurements was largest, −70 mL, for pair (1,2) and insignificant for the other pairs (Figure S2b,d,f). BA plots obtained for each sex in consecutive pairs of trials are shown in Figure S3 for FFM and Figure S4 for BV.

Absolute reliability parameters computed for successive pairs of ADP tests are listed in Table 2. They are expressed in the same units as the corresponding body composition variable. Small values of these parameters indicate high reliability.

Table 3 presents relative measures of reliability; these are dimensionless quantities ranging from 0 to 1 (the higher, the better).

The correlation coefficients for body volume measurements are not included in Table 3 because they were extremely high—0.9999 for pair (1,2) and 1.0 for the other two pairs.

Since the reliability estimates calculated from pairs (2,3) and (3,4) were similar (Table 2 and Table 3), we dumped together the last triplet of trials (tests 2, 3, and 4) to compute more precise measures of the reliability of individual ADP trials; these are listed in Table 4.

Table 4 suggests that once the subject is acquainted with the test procedure, the reliability of single trials is comparable to that of multiple measurement protocols, such as the one proposed by Tucker et al. [47] (henceforth called the Tucker protocol), or the Median protocol [48]. The Tucker protocol asks for at least two consecutive trials. If these are within 1% BF, their mean is computed; otherwise, a third trial is performed and the mean of the two closest readings is taken as the measurement result. The Median protocol consists of taking the median of triplicate assessments.

The BA plots from Figure S5 indicate that %BF values from the second and fourth trials did not differ on average from the Tucker protocol or the Median protocol. As expected, the intervals of agreement were narrower for the second reading, which is part of the triplet involved in those protocols (Figure S5a–d). The first assessment, however, showed a significant bias, of about −0.6 %BF, compared to the results of both protocols computed from assessments 2 to 4 (Figure S5e,f).

## 4. Discussion

In this paper, we conducted quadruplicate ADP tests on a heterogeneous group of adults with no previous experience with ADP. Several methods of statistical analysis confirmed our working hypothesis that learning effects can indeed affect body composition assessments by ADP. The reported results suggest that the first-ever ADP test is prone to underestimating the subject’s adiposity. Although statistically insignificant according to the Kruskal–Wallis test, this underestimation is about half of the technical error of ADP measurements (see Figure 2a and Table 2).

The learning effects observed in this study might explain the wide range of typical errors estimated in previous investigations of the BOD POD’s reliability: the TEM was 0.55% BF in the study carried out by Peeters on 25 male subjects of about 20 years of age [35], 0.57% BF in the work of Peeters and Claessens involving 31 women and 31 men in their early twenties [49], 0.80% BF in the paper of Collins and McCarthy on 57 women and 45 men aged between 15 and 55 y [23], 1.07% BF in the study of Noreen and Lemon involving 432 women and 548 men aged 30 ± 15 years, and 1.28% BF in the study performed by Anderson on 16 women and 8 men between 18 and 38 years of age [21]. In the present study, the TEM computed from the first two trials was 1.04% BF, similar to the one observed by Noreen and Lemon, but it dropped to 0.71% BF when the calculation was done from the second pair of readings (Table 2). Hence, differences between the TEM values reported to date could stem from differences in the familiarity of the subjects with ADP.

The negative bias observed in the present study between the first two trials was not observed in previous investigations of the reliability of ADP. Both the Bland–Altman analysis of [23] and the statistical analysis of [27] indicated a good agreement between the mean %BF assessments provided by the first two trials. The reason for this disagreement is not clear, but our focus on novice subjects might have played a role in this respect.

ADP reliability estimates can also be influenced by occasional aberrant body composition assessments given by this technique. First reported by Wells and Fuller [50], such rogue values are identified when the discrepancy between successive measurements exceeds a cutoff value of about 3% BF. Their origin is still unknown. In the large-scale study of Noreen and Lemon [27], aberrant values were spotted in 32 of the 980 participants. They argued that, whatever the cause of rogue results might be, it should last for at least 3–5 min to affect the second or third body volume measurements involved in one ADP test. In the present study, we defined an aberrant assessment as one that differed by at least 3% BF from the average of the closest two %BF values out of all four trials. We observed seven rogue assessments and five of them originated from the first trial, one from the second, and one from the fourth. We did not observe two aberrant readings on the same subject. Although the low number of observations hampers statistical reasoning, it seems safe to conclude that aberrant results mainly stem from the first measurement; thus, it is advisable to discard it. Then, the question is how to proceed in the rare cases when the second reading differs by more than 3% BF from the first one (i.e., one of them is rogue). Then, a third assessment is needed, and one can apply a multiple-assessment protocol to assign a result based on all three measurements. According to the Tucker protocol [47], the final result would be the mean of the two closest readings; according to the Median protocol [48], it would be the median of the three readings. While other options for computing the final result are also available, their reliabilities need to be evaluated in future investigations.

Since the reliability indices settled down starting from the second trial, we pooled tests 2 to 4 together for a more precise calculation of reliability indices [32]. Remarkably, once the first test was discarded, single measures were almost as reliable as multiple measurement protocols [48]. For instance, for %BF measurements, ICC(2,1) was 0.9959 for the Tucker protocol and 0.9967 for the Median protocol, whereas this study gave 0.9960 for single tests. The TEM and SEM were both 0.70% BF for the Tucker protocol and 0.62% BF for the Median protocol, similar to the SEM obtained here for single trials, 0.70% BF. A recent study of the reliability of ADP in the Indian male population [51] reported a significantly lower SEM, 0.44% BF, but its estimate was based on pairs of trials averaged in the course of one Tucker procedure [47]. It remains to be established whether the SEM remains equally low when it is computed from the test and retest results, both of them being provided by an entire Tucker protocol.

Finally, for the first time, according to our knowledge, this study reports the minimum change in body composition detectable by ADP in the absence of learning effects (Table 4).

The main limitations of the present study are (i) its exclusive focus on novice subjects and (ii) its methodology based on same-day, contiguous tests. Thus, it is unable to answer the question of whether returning participants would also need a practice trial before the actual measurement. Furthermore, how much time can pass before familiarity is lost? These questions deserve further scrutiny, although Anderson’s work [21] sheds some light on them. On his sample of 24 adults, he performed pairs of trials on three different days within a period of one week. The TEM was 1.15% BF on Day 1 and, surprisingly, 1.28% BF on Days 2 and 3, suggesting that the practice of Day 1 faded away within a few days.

The mechanism responsible for the marginal underestimation of global adiposity by the first ADP test is unclear. It is known, however, that a new experience can lead to anticipatory anxiety, which results in increased respiratory frequency and expired ventilation [52]. Furthermore, changes in the subject’s breathing pattern are known to affect %BF assessment by ADP [53]. Future investigations, with intentionally triggered anticipatory anxiety, along the lines devised by Masaoka and Homma [52], might elucidate the cause of learning effects and rogue results in ADP.

Nevertheless, it is worth noting that none of our participants complained or mentioned anxiety associated with ADP tests.

## 5. Conclusions

This study demonstrated that learning effects may play a significant role in body composition assessments by ADP. Regardless of sex, consecutive tests became increasingly concordant, presumably because the subjects grew accustomed to the test procedure and, consequently, their breathing pattern settled down.

Both absolute and relative indices of reliability leveled off starting from the second pair of trials, becoming comparable to those of multiple-assessment protocols. Therefore, the present study suggests that, at least for novice subjects, the first trial should be considered a practice test. Then, the result of the second trial can be accepted if it differs by less than 3% BF from the first one; otherwise, a third test is needed, and the body composition variables of the subject can be computed using a repeated trials protocol.

## Figures and Tables

**Table 1:** Demographic data of the study sample—mean ± standard deviation (SD), range, and median enclosed in brackets.

	All (*n* = 105)	Women (*n* = 51)	Men (*n* = 54)
Age (y)	30.4 ± 10.2 [20.0, 66.5] (25.1)	31.2 ± 12.6 [20.0, 66.5] (24.6)	29.6 ± 7.3 [20.3, 54.9] (26.3)
Height (m)	1.71 ± 0.10 [1.49, 1.92] (1.71)	1.63 ± 0.06 [1.49, 1.77] (1.63)	1.80 ± 0.06 [1.69, 1.92] (1.80)
BM ^1^ (kg)	71.7 ± 16.9 [38.0, 156.0] (72.2)	62.4 ± 12.0 [38.0, 94.4] (59.2)	80.4 ± 16.2 [57.5, 156.0] (78.2)
BMI (kg/m^2^)	24.2 ± 4.5 [16.7, 45.1] (23.9)	23.6 ± 4.5 [16.7, 33.7] (21.9)	24.9 ± 4.3 [17.7, 45.1] (24.6)

^1^ Abbreviation: BM—body mass; BMI—body mass index.

**Table 2:** Absolute indices of reliability computed for consecutive pairs of trials.

		All (*n* = 105)			Women (*n* = 51)			Men (*n* = 54)		
Variable	Pair	TEM ^1^	SEM	MDC	TEM	SEM	MDC	TEM	SEM	MDC
%BF (%)	(1,2)	1.04	1.04	2.88	1.10	1.10	3.04	0.98	0.98	2.71
	(2,3)	0.71	0.71	1.97	0.80	0.80	2.21	0.61	0.61	1.69
	(3,4)	0.66	0.66	1.82	0.72	0.72	1.99	0.59	0.59	1.64
FFM (kg)	(1,2)	0.730	0.728	2.018	0.671	0.668	1.851	0.781	0.777	2.154
	(2,3)	0.511	0.510	1.412	0.500	0.498	1.380	0.521	0.518	1.436
	(3,4)	0.452	0.451	1.249	0.422	0.420	1.165	0.478	0.476	1.319
BV (L)	(1,2)	0.146	0.145	0.403	0.134	0.133	0.369	0.156	0.155	0.431
	(2,3)	0.103	0.102	0.284	0.102	0.101	0.281	0.103	0.103	0.285
	(3,4)	0.107	0.107	0.296	0.084	0.084	0.233	0.125	0.124	0.344

^1^ Abbreviation: TEM—technical error of measurement; SEM—standard error of measurement; MDC—minimum detectable change.

**Table 3:** Relative measures of reliability computed for consecutive pairs of trials.

		All (*n* = 105)		Women (*n* = 51)		Men (*n* = 54)	
Variable	Pair	ICC (2,1) ^1^	CCC	ICC (2,1)	CCC	ICC (2,1)	CCC
%BF (%)	(1,2)	0.9910	0.9909	0.9884	0.9862	0.9871	0.9868
	(2,3)	0.9958	0.9958	0.9927	0.9927	0.9950	0.9949
	(3,4)	0.9964	0.9964	0.9943	0.9942	0.9953	0.9953
FFM (kg)	(1,2)	0.9971	0.9971	0.9881	0.9878	0.9914	0.9913
	(2,3)	0.9986	0.9986	0.9937	0.9935	0.9961	0.9961
	(3,4)	0.9989	0.9989	0.9955	0.9954	0.9967	0.9966

^1^ Abbreviation: ICC (2,1)—2-way random effects model intraclass correlation coefficient [43]; CCC—Lin’s concordance correlation coefficient [44].

**Table 4:** Test-retest reliability indices of single ADP tests. These were computed from triplicate measurements conducted after the first trial—regarded as a practice test.

	All (*n* = 105)			Women (*n* = 51)			Men (*n* = 54)		
Variable	ICC (2,1)	SEM	MDC	ICC (2,1)	SEM	MDC	ICC (2,1)	SEM	MDC
%BF (%)	0.9960	0.70	1.93	0.9933	0.77	2.14	0.9950	0.61	1.68
FFM (kg)	0.9987	0.49	1.35	0.9943	0.47	1.30	0.9963	0.50	1.39
BV (L)	1.0000	0.107	0.297	0.9999	0.095	0.264	1.0000	0.117	0.323

## Data Availability

Data acquired during this study have been anonymized and shared in Supplementary Materials.

## Supplementary Materials

The following are available online at https://www.mdpi.com/article/10.3390/life13061315/s1; Table S1: *p*-values returned by the Shapiro–Wilk test for normal distribution; Table S2: *p*-values returned by the Kruskal–Wallis test; Table S3: Absolute indices of reliability computed for successive pairs of trials performed on participants other than the first subject tested in any given day of measurements; Figure S1: Violin plots of body volumes measured in 4 consecutive ADP tests; Figure S2: BA plots of differences vs. means of FFM and BV assessments in 3 pairs of consecutive trials; Figure S3: BA plots illustrating learning effects in FFM assessments as a function of sex; Figure S4: BA plots of differences vs. means of body volumes obtained in successive pairs of measurements conducted on women (panels (a,c,e)), and men (panels (b,d,f)); Figure S5: BA analysis of single tests compared with protocols based on multiple trials; Data S1: Anonymized data file.
