# Supplementary material for: Learning Effects in Air Displacement Plethysmography
Source: Life (Basel). 2023 Jun 2;13(6):1315. doi: 10.3390/life13061315 (PMC10304159; doi:10.3390/life13061315)
Supplement: Supplementary file 1 [file life-13-01315-s001.zip › ADP_learning_SupplementaryMaterials.pdf]

life

MDPI

Supplementary Materials

for the Article

Learning Effects in Air Displacement Plethysmography

Paul Muntean, Anca Popa, Monica Miclos-Balica, Falk Schick, Oana Munteanu, Vasile Pupazan, Adrian Neagu, and Monica Neagu

This document presents supplementary illustrations. Although their captions are supposed to render them intelligible, their relevance in the context of this study is only explained in the main text of the article.

Table S1. *P*-values returned by the Shapiro-Wilk test for normal distribution

Variable	All (*n* = 105)	Women (*n* = 51)	Men (*n* = 54)
%BF	$< 10^{-4}$	$< 10^{-4}$	2×10^{-4}
FFM (kg)	$< 10^{-4}$	$< 10^{-4}$	$< 10^{-4}$
BV (L)	$< 10^{-4}$	$< 10^{-4}$	$< 10^{-4}$

Abbreviation: %BF – body fat percentage; FFM – fat-free mass; BV – body volume.

Table S2. *P*-values returned by the Kruskal-Wallis test

Variable	All (*n* = 105)	Women (*n* = 51)	Men (*n* = 54)
%BF	0.9688	0.9712	0.9881
FFM (kg)	0.9892	0.9486	0.9838
BV (L)	0.9997	0.9997	0.9994

Table S3. Absolute indices of reliability computed for successive pairs of trials performed on participants other than the first subject tested in any given day of measurements – denoted by * in Figure 2 b, d, and f.

		All (*n** = 67)			Women (*n** = 30)			Men (*n** = 37)		
Variable	Pair	TEM[1]	SEM	MDC	TEM	SEM	MDC	TEM	SEM	MDC
%BF (%)	(1,2)	1.00	1.00	2.76	1.03	1.02	2.84	0.97	0.97	2.68
	(2,3)	0.72	0.72	2.00	0.82	0.82	2.26	0.63	0.63	1.74
	(3,4)	0.68	0.68	1.89	0.80	0.79	2.20	0.57	0.57	1.58
FFM (kg)	(1,2)	0.715	0.712	1.973	0.653	0.647	1.792	0.761	0.756	2.095
	(2,3)	0.531	0.529	1.467	0.509	0.505	1.400	0.549	0.545	1.510
	(3,4)	0.472	0.471	1.304	0.469	0.465	1.290	0.475	0.471	1.307
BV (L)	(1,2)	0.145	0.145	0.401	0.133	0.132	0.366	0.154	0.153	0.425
	(2,3)	0.107	0.107	0.296	0.105	0.104	0.288	0.109	0.108	0.301
	(3,4)	0.095	0.095	0.263	0.094	0.093	0.257	0.096	0.096	0.265

[1] Abbreviation: TEM – technical error of measurement; SEM – standard error of measurement; MDC – minimum detectable change.

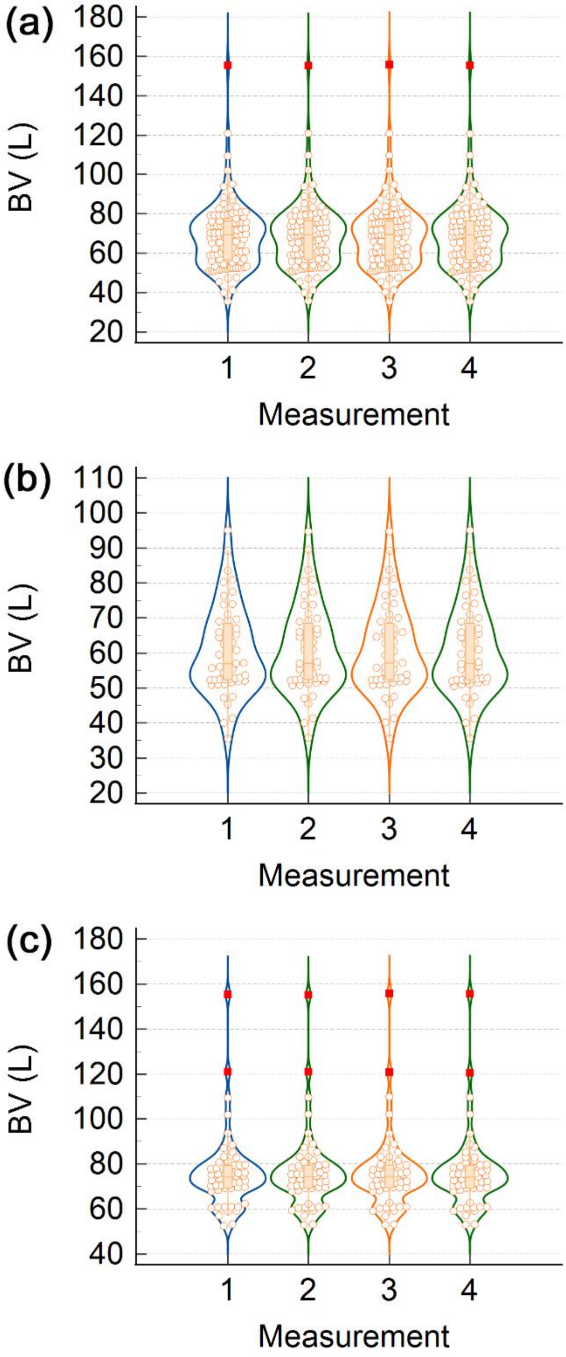

Figure S1. Violin plots of body volumes measured in 4 consecutive ADP tests. Shown are (**a**) the body volume (BV) of all participants, (**b**) BV of women, and (**c**) BV of men.

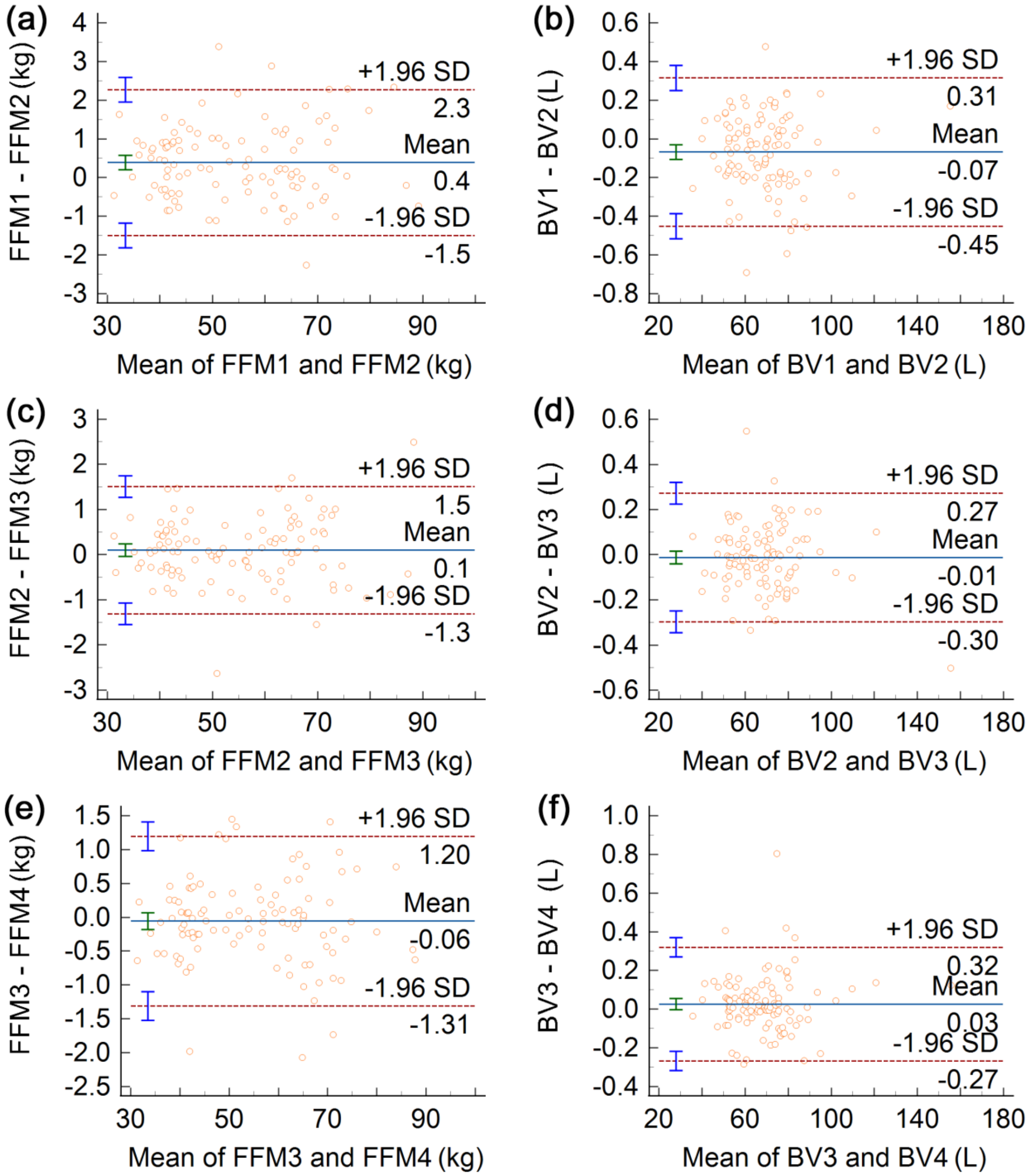

Figure S2. BA plots of differences vs. means of FFM and BV assessments in 3 pairs of consecutive trials. The first two test results are compared in (**a**) and (**b**), the next two are compared in (**c**) and (**d**), and the last two are compared in (**e**) and (**f**). Panels (**a**), (**c**), and (**e**) refer to FFM, whereas (**b**), (**d**) and (**f**) refer to BV.

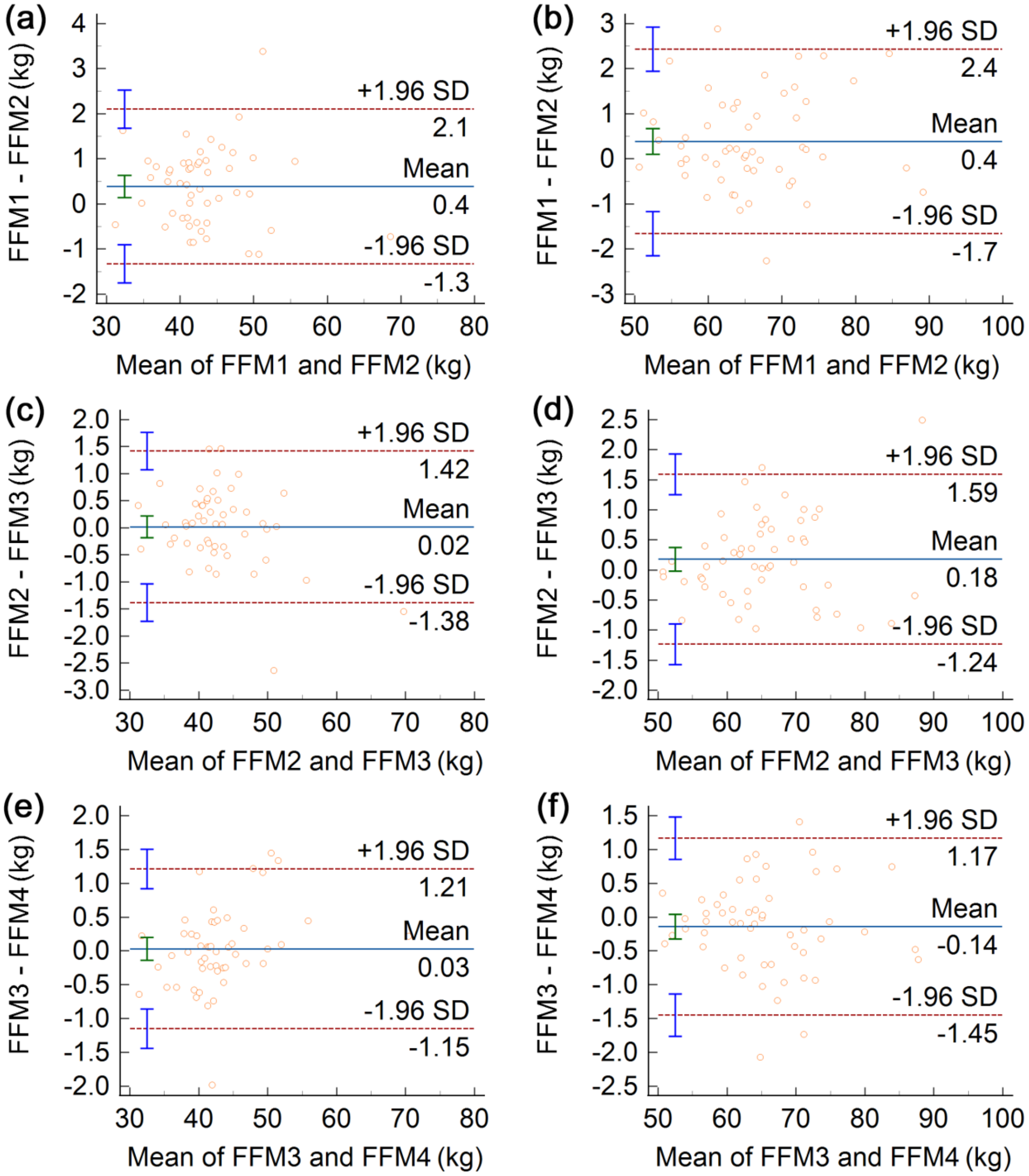

Figure S3. BA plots illustrating learning effects in FFM assessments as a function of sex. The first pair of measurements is analyzed in panels (**a**) and (**b**), the second in (**c**) and (**d**), and the third in (**e**) and (**f**). Panels (**a**), (**c**), and (**e**) refer to women, whereas (**b**), (**d**), and (**f**) correspond to men.

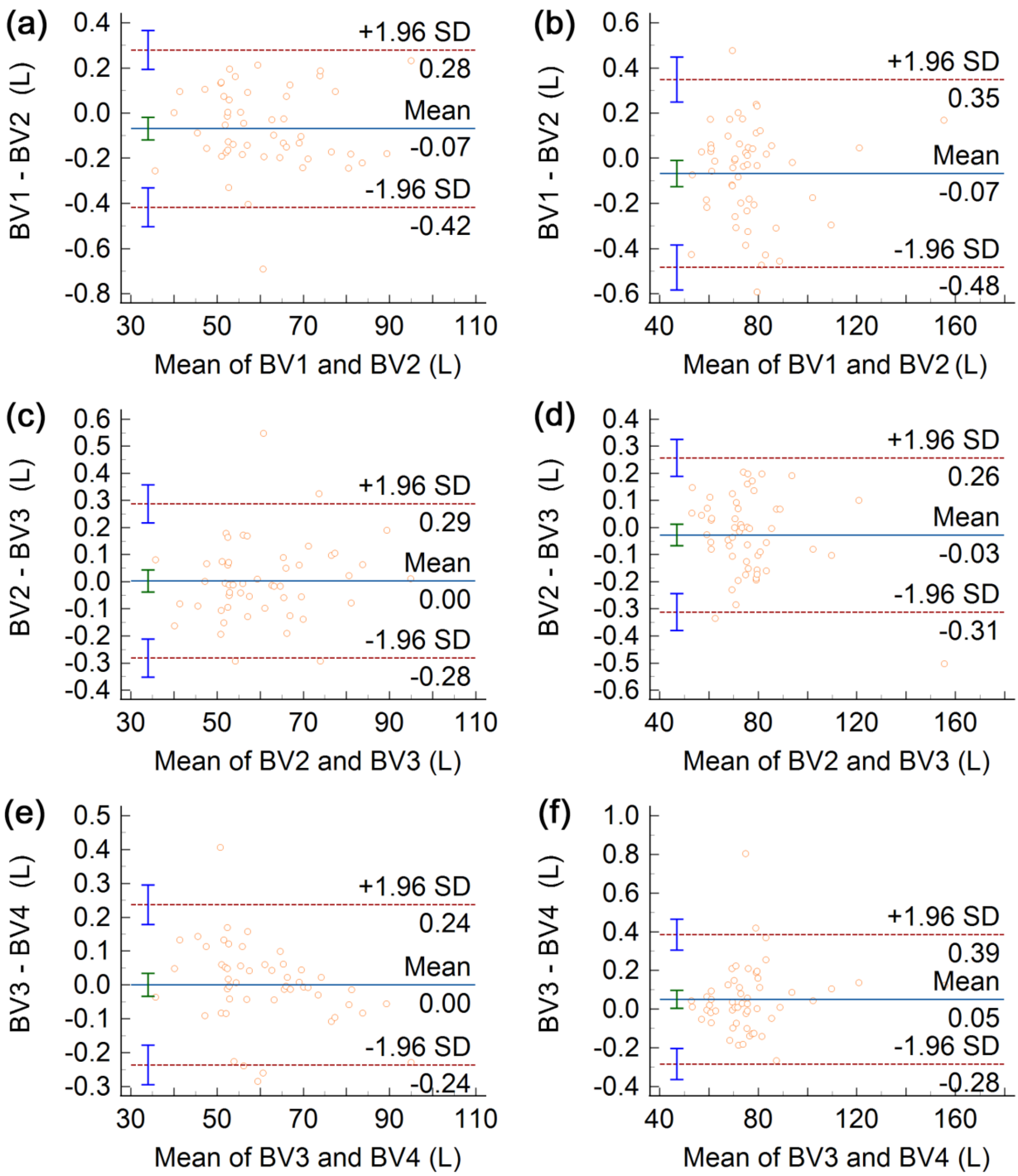

Figure S4. BA plots of differences vs. means of body volumes obtained in successive pairs of measurements conducted on women (panels (**a**), (**c**), and (**e**)), and men (panels (**b**), (**d**), and (**f**)). The first two test results are compared in panels (**a**) and (**b**), the next two are compared in (**c**) and (**d**), whereas the last two are compared in (**e**) and (**f**).

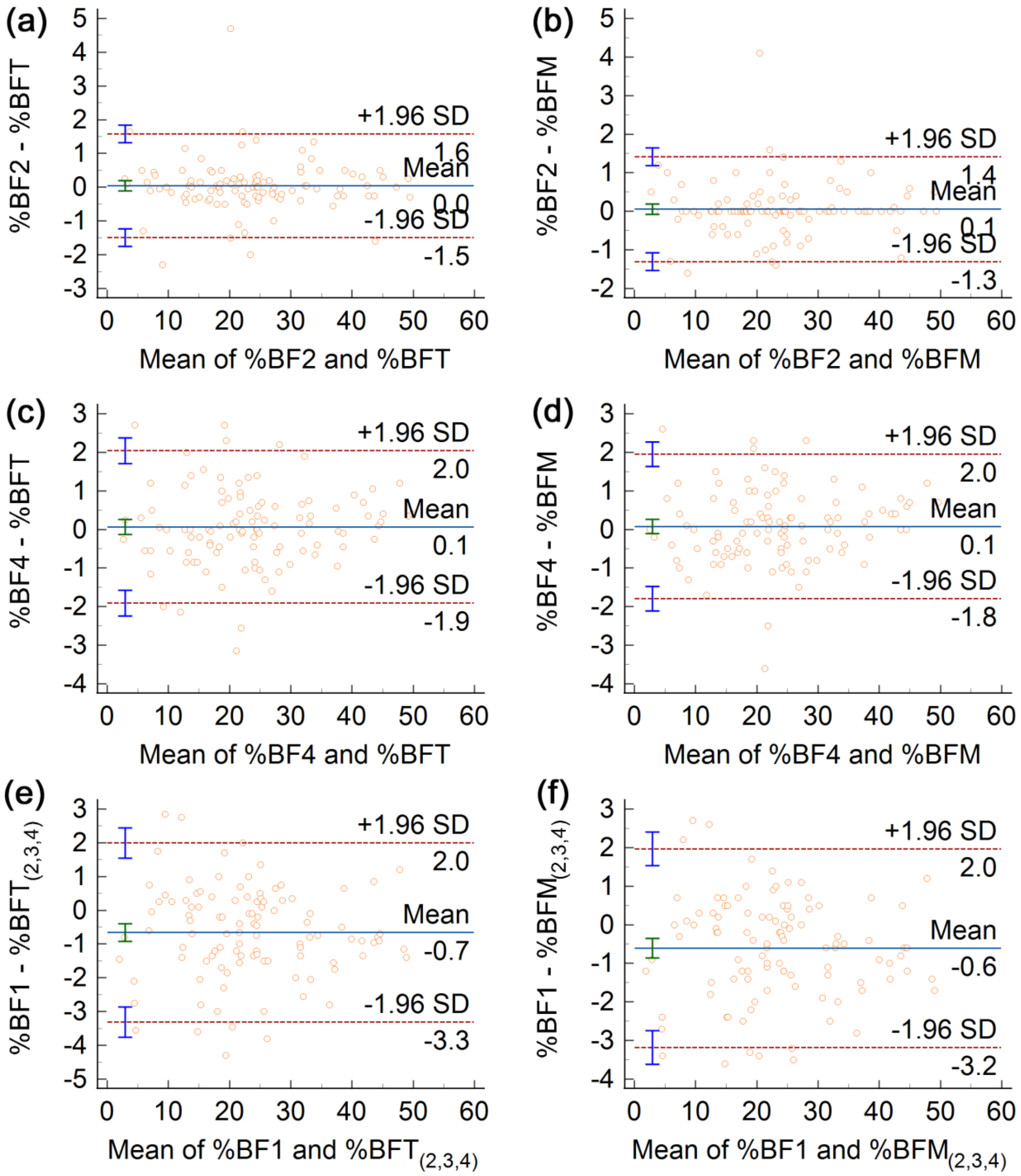

Figure S5. BA analysis of single tests compared with protocols based on multiple trials. Here, %BFT denotes %BF assessment according to the multiple measurements procedure proposed by Tucker et al. [47] (hereafter called Tucker protocol), whereas %BFM stands for %BF obtained using the Median protocol (i.e. taking the median of three consecutive assessments) [34]. Both of these involve the first three trials; %BFT (2,3,4) and %BFM (2,3,4) refer to similar variables extracted from the last three trials (i.e. when the first one is discarded as a practice test). (**a**) %BF2 compared with %BFT, (**b**) %BF2 compared with %BFM (**c**) %BF4 compared with %BFT; (**d**) %BF4 compared with %BFM, (**e**) %BF1 compared with the Tucker protocol applied for trials 2, 3, and 4, and (**f**) %BF1 compared with the median of trials 2, 3, and 4.
